# Supplementary figures and images for: Whole Genome Analysis of SLs Pathway Genes and Functional Characterization of DlSMXL6 in Longan Early Somatic Embryo Development
Source: Int J Mol Sci. 2022 Nov 14;23(22):14047. doi: 10.3390/ijms232214047 (PMC9695034; doi:10.3390/ijms232214047)

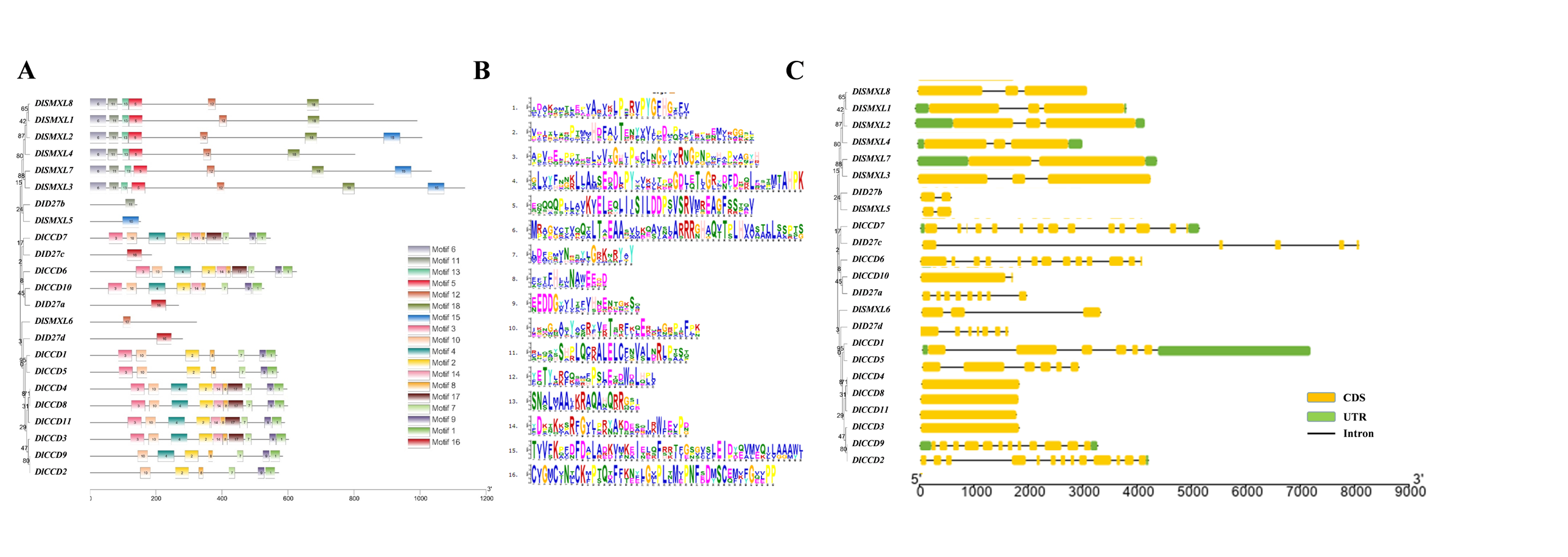

Supplement: Supplementary file 1 [file ijms-23-14047-s001.zip › Figure S1.tif]

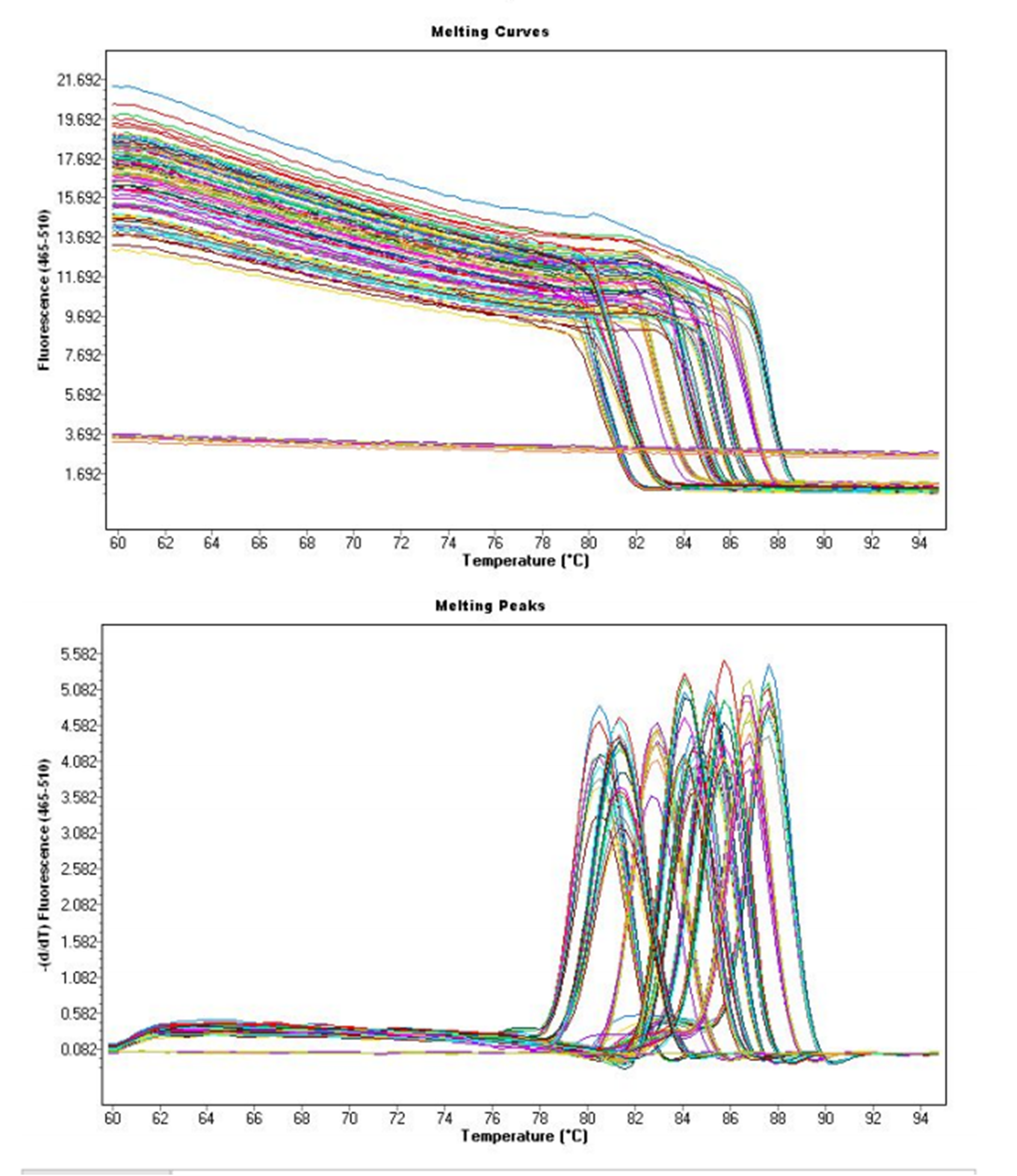

Supplement: Supplementary file 1 [file ijms-23-14047-s001.zip › Figure S2.tif]
